# Novel clinical therapeutics targeting the epithelial to mesenchymal transition

**DOI:** 10.1186/s40169-014-0035-0

**Published:** 2014-10-15

**Authors:** Anai N Kothari, Zhiyong Mi, Matthew Zapf, Paul C Kuo

**Affiliations:** 1 Department of Surgery, Oncology Institute, Loyola University Medical Center, 2160 South First Ave, EMS Bldg, Rm 3244, Maywood 60153, IL, USA

**Keywords:** Epithelial mesenchymal transition (EMT), Small molecule inhibitors, TGFβ, Cancer associated fibroblasts

## Abstract

The epithelial to mesenchymal transition (EMT) is implicated in many processes, ranging from tissue and organogenesis to cancer and metastatic spread. Understanding the key regulatory mechanisms and mediators within this process offers the opportunity to develop novel therapeutics with broad clinical applicability. To date, several components of EMT already are targeted using pharmacologic agents in fibrosis and cancer. As our knowledge of EMT continues to grow, the potential for novel therapeutics will also increase. This review focuses on the role of EMT both as a necessary part of development and a key player in disease progression, specifically the similarity in pathways used during both processes as targets for drug development. Also, the key role of the tumor microenvironment with EMT is outlined, focusing on both co-factors and cell types with the ability to modulate the progression of EMT in cancer and metastatic disease. Lastly, we discuss the current status of clinical therapies both in development and those progressed to clinical trial specifically targeting pathologic EMTs including small molecule inhibitors, non-coding RNAs, exogenous co-factors, and adjunctive therapies to current chemotherapeutics.

## 1 Introduction

The epithelial to mesenchymal transition (EMT) was first described in the context of being a fundamental component in the differentiation of tissues and organs within the developing embryo [1]. Beyond tissue and organogenesis, EMT has been described as a crucial mediator of wound healing, a feature of EMT necessary for tissue remodeling post-injury [2]. The characteristics of EMT that are essential to wound healing also connect EMT to organ fibrosis, including pulmonary fibrosis, renal fibrosis, and hepatic fibrosis. In these settings, EMT is intimately linked to diseased states. The integral role of EMT in disease was taken one step further with evidence supporting a key role in tumor initiation and, subsequently, the progression of metastatic disease [3].

EMT is contemporarily classified into 3 classes: type 1 referring to embryogenesis; type 2 referring to wound healing; type 3 referring to its role in cancer and metastases. A hallmark of each is the process through which epithelial cells lose their cell-cell junctions, apical-basal polarity, and re-organize their cytoskeleton by e-configuring their gene expression. These processes are highly regulated in the setting of type 1 and 2 EMT. In type 3 EMT, dysregulation leads to diseased states [4].

On a molecular level, EMT is defined by several intermediates responsible for influencing gene expression. These include transcription factors, multiple interacting signal pathways, promoter sequence modifications, and autocrine signaling. Of these, the principal regulators of EMT are transcription factors classified into 3 families: SNAIL, TWIST, and zinc-finger E-box-binding (ZEB). The fundamental event in EMT is the repression of epithelial cadherin (E-cadherin), which is responsible for maintaining adherens junctions and cell-cell adhesion. SNAIL, TWIST, and ZEB factors all can repress E-cadherin while also activating key mesenchymal genes including N-cadherin (cell-cell adhesion), vimentin (an intermediate filament protein that regulates cell motility), and fibronectin 1 (cell growth and migration). Together, these transcription factors orchestrate the activation of mesenchymal gene expression while repressing epithelial genes through diverse downstream targets.

**Figure 1:**
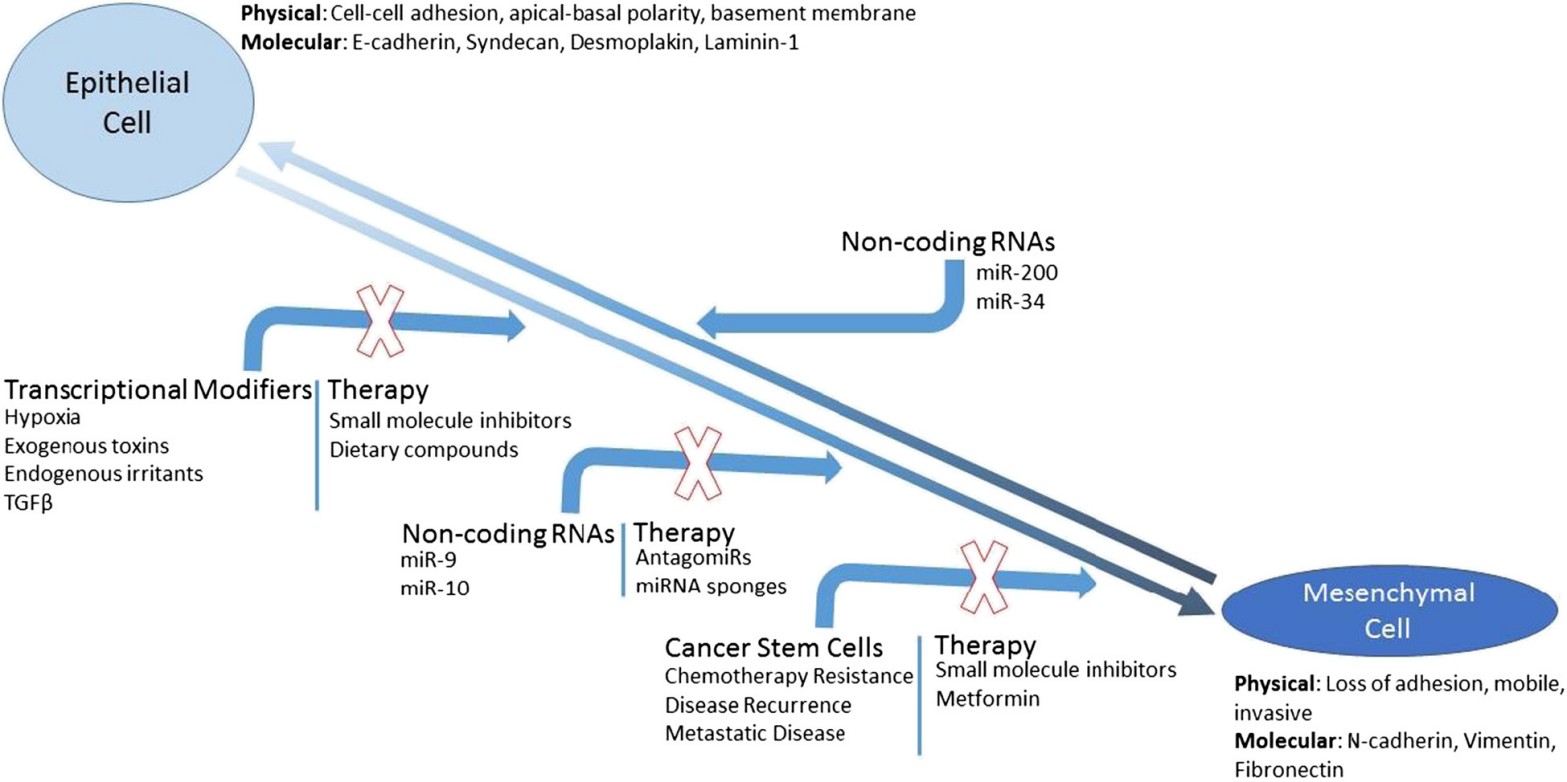
Schematic of EMT, EMT inducers, and potential or existing areas within the framework for therapeutic intervention.

The same mediators used for modifying gene expression are used as biomarkers to identify a cell as epithelial or mesenchymal. For example, expression of E-cadherin is used to describe a cell as being in an epithelial state whereas expression of N-cadherin, vimentin, and/or fibronectin 1 are used to describe the mesenchymal phenotype (see Figure 1). The importance and clinical relevance of these pathways and mediators will be discussed throughout this review [5].

The focus of this review will be on 3 major areas. First, the role of EMT as a necessary pathway to development and healing. Next, the pathophysiologic impact of EMT on diseased states (primarily fibrotic phenotypes and cancer). Finally, discussing the areas within the spectrum of EMT which allow for novel therapeutics and the current progress towards clinical application.

## 2 Review

### 2.1 SECTION 1: The role of EMT in development and diseased states

#### 2.1.1 EMT and development

EMTs are pathways followed during normal development; most organs and adult tissues are generated via these transitions embryologically. Each of these transitions rely on signal cascades with multiple regulatory and feedback loops allowing both plasticity and cooperation during development. EMT plays a prominent role during gastrulation and many of the key molecular players during these processes have been noted to be important in disease progression during adulthood. For example, members of the TGFβ superfamily are powerful inductors of gastrulation [6]. SNAIL1 and SNAIL2, known mediators of EMT in adults, are dependent on TGFβ activation and required for gastrulation to proceed [7],[8].

The importance of key EMT mediators in early gastrulation is further emphasized in SNAIL-mutant mice where cells are unable to migrate from the primitive streak, effectively halting development [9]. TGFβ signaling and the SNAIL family of transcription factors are two important influences during dysregulated EMT in adult disease, as well. Understanding the signaling targets during embryologic EMT and comparing them to those found in the progression of disease is an area of research that may offer new therapeutic targets.

#### 2.1.2 EMT and diseased states

EMT has a role in several disease states including pathologic organ degradation (ie fibrosis) and cancer. How EMT fits into these processes will be reviewed, including the initiating signal pathways, downstream targets, and mechanisms for regulating these pathologic EMTs.

In all pathologic EMTs, a series of steps are conserved and are crucial for defining an epithelial cell’s conversion to a mesenchymal phenotype. Epithelial cells are broadly characterized as those positioned on the basement membrane (either as single layered or multilayered tissues), show polarity, adhere, and communicate using cell-cell junctions [10]. Loss of these characteristics places epithelial cells on a spectrum. On one end is the epithelial phenotype and on the other end is the mesenchymal phenotype. At the furthest extreme, the mesenchymal phenotype, the result is cells which are invasive and resistant to apoptosis [11]. These features of EMT are consistent between cell types and tissue environments.

One such example can be seen in the pathways regulating cell-cell junctions and their dissolution during EMT. Cell-cell junctions are critical for maintaining basement membranes and the integrity of epithelial layers. In vertebral cells, tight junctions, gap junctions, and desmosomes are the primary regulators of these connections [11],[12]. Several EMT activators act either jointly or independently on each of these cell contact mediators and their impact on cell-cell junctions is nearly the same regardless of cell type [5]. The importance of factors on the cell surface to EMT is further illustrated by recent findings illustrating that alterations in the glycosylation of cell surface glycoproteins impacts cellular adhesive properties. The characteristics of altered O- and N-glycans through mRNA expression during tumor progression is extremely relevant and well described in a recent review [13].

##### EMT and organ fibrosis

Fibrotic tissues are characterized by an up-regulation of extracellular matrix (ECM) protein deposition eventually leading to compromise of organ function, and in the most severe cases, organ failure [14]. The cells responsible for ECM protein deposition are myofibroblasts typically in response to epithelial or endothelial injury [15]. As tissue repair progresses, the role of myofibroblasts shifts from creating a scaffold for healing into facilitating wound contraction and closure [16]. Myofibroblasts originate from several cell types most notably existing epithelial cells, endothelial cells, interstitial fibroblasts, and myeloid precursor cells in bone marrow [17]-[20].

Inflammatory cytokines, specifically TGFβ and IL-1, are critical in the differentiation of cells from any site into myofibroblasts [21]. In wound healing, myofibroblasts are key to creating wound tension and depositing important co-factors required for wound contraction. This process is tightly regulated, and at its completion, the population of myofibroblasts recruited to the site of injury to promote healing undergoes population-wide apoptosis [22]. In situations where myofibroblasts persist in the extracellular matrix, a fibrotic phenotype develops. In wounds, this is manifested as hypertrophic scar or keloid [23]. In organs, the result is fibrosis [24],[25].

Alveolar epithelial cells, hepatocytes, lens epithelium, endothelial cells, mesothelial cells, and cardiomyocytes all have the potential to undergo EMT and resemble myofibroblasts, subsequently contributing to organ fibrosis [26]. In pulmonary fibrosis models, TGFβ stimulates EMT in alveolar epithelial cells. Similarly, CCL4-induced liver fibrosis is linked to EMT in hepatocytes. Recent evidence shows a role for EMT in peritoneal fibrosis in patients that receive peritoneal dialysis in a MAPK and SNAIL1 dependent fashion [27]. EMT in lens epithelial cells causes progressive capsular opacification and can be prevented by inhibiting TGFβ with Smad7 [26].

In renal fibrosis, mice that do not express Smad3 (a target of TGFβ) are protected from development of fibrosis. The crucial role of TGFβ in renal fibrosis was taken a step further by Zeisberg et al. who demonstrated that inhibiting TGFβ with an antagonist (BMP7) actually could revert renal fibrosis in mice [28].

##### EMT and cancer

The pathologic potential of EMT is seen in organ fibrosis and similar mechanisms are seen in the development of cancer, evolution of disease, and metastatic progression. In cancer, EMTs rely on: 1) exogenous induction agents acting through transcriptional control 2) Non-coding RNAs and 3) creating and maintaining cancer stem cells [29].

1. Transcriptional control mechanisms

The discovery of the transcription factor SNAIL1 interacting with the CDH1 promoter to repress the production of E-cadherin began our understanding of how EMT is regulated [30],[31]. Numerous other transcription factors have been found to play a role in the progression of epithelial cells to a mesenchymal phenotype including SNAIL2, ZEB1, ZEB2, E47, KLF8, TWIST1, and FOXC2, all which have the ability to promote EMT in various cell cancer lines [32],[33]. Of these, the nuclear factors which seem to be most important are SNAIL, ZEB, and TWIST proteins and these can be modulated to initiate EMT by exogenous stimuli including hypoxia, bile acids, and nicotine [34]-[36].

The association between disease progression and solid tumor oxygenation is seen in various malignancies. It is apparent that tumor hypoxia contributes to several features of aggressive cancers by enhancing invasive growth, local spread, and distant deposits [37]. The mechanism for hypoxic influence of these features is likely multifactorial, however there is clear interplay with EMT. In pancreatic tumor cells, hypoxia (induced by culturing cells in a hypoxic chamber) resulted in expression of EMT makers when compared to normal conditions [34]. This observation is seen in other solid tumors including lung and breast [38].

Cholestatic disorders seem to promote hepatic fibrosis and to the progression of carcinogenesis in cholangiocytes and hepatocytes. Studies have shown that chendeocycholic acid (CDCA), a bile acid, can activate EGFR to mediate gene expression. In a cDNA microarray, CDCA increased the expression of SNAIL to repress E-cadherin both in vitro and in vivo in liver cancer models, initiating an EMT program [36]. The ability for bile acids to play an activating role is just one of many examples where exogenous substrates can induce EMT.

Another substrate which activates EMT through a similar mechanism is nicotine. Specifically, in breast and lung cancer cell lines (A549, MDA-MB-468, MCF-7), treatment with nicotine increased the proliferation, invasion, and migration of both lung and breast cancer cells in a dose-dependent fashion. Notably, expression of E-cadherin was repressed while the expression of fibronectin and vimentin, both mesenchymal markers, increased. The mechanism for these changes occurred through signaling by nicotinic acetylcholine receptors (nAChRs) [35].

Recent data demonstrates a pathway for cigarette smoke to stimulate EMT in small bronchi of patients demonstrating a mechanism contributing to the progression of disease. In primary human bronchial epithelial cells (HBEC), exposure to cigarette smoke led to expression of EMT markers by increased production of intracellular ROS, release of TGFβ, and TGFβ activation of the transcription factors Smad3 and ERK1/2 [39]. This kind of interaction between an exogenous substrate and the cellular machinery activating EMT has significant clinical implication as treatments can be designed to potentially halt or reverse the progression of disease.

2. Non-coding RNAs

Non-coding RNAs and, in particular, microRNAs (miRNAs) have gained significant attention for their ability to modify gene expression allowing for quick cellular adaptation to new environments and conditions [29],[40]. The regulatory association of miRNAs is seen in many settings including development, maintaining cell homeostasis, and cell-cell interaction [41]. Li et al., in 2009, showed a role for miRNAs in reversing EMT in pancreatic tumor cells that were resistant to common chemotherapeutics [42].

Since that time, numerous miRNAs have been found to interact either directly or indirectly with EMT machinery in several cell models [43]. Interestingly, these miRNAs can act as either tumor suppressors or tumor activators based on the cell type and environment they interact with. Additionally, their adaptive properties play a role in chemotherapy resistance.

Two major classes of miRNAs have been studied extensively in the setting of EMT: the miR-200 family and the miR-34 family. Other families include the miR-205, miR-9, and miR-10 families, respectively. The miR-200 family has five named members located on human chromosome 1 (mir-200a, mir-200b, mir-429) and 12 (mir-200c, mir-141). Both the miR-200 and miR-34 families are typically considered to be “tumor suppressive” as their members all stabilize the epithelial phenotype [44].

The interplay between miR-200 and the ZEB family of transcription factors to maintain E-cadherin expression has been validated in several systems. In a TGFβ-induced model of EMT in a murine mammary cell line, miR-200 family member expression was repressed. This same cell line was transfected with synthetic pre-miRNAs to stimulate overexpression of corresponding miR-200 members in vitro. The result was inhibiting EMT in the transfected cell lines, when compared to control, by increasing the expression of E-cadherin. Furthermore, using an elegantly designed luciferase assay, miR-200 family members were noted to interact directly with the transcription factors ZEB1 and ZEB2 [45]. ZEB1 and ZEB2 typically act as EMT inducers by suppressing E-cadherin expression, however in the presence of miR-200, ZEB1 and ZEB2 could not interact with E-cadherin and the epithelial phenotype was maintained [45],[46].

The clinical relevance of miR-200 family members can be seen in malignant breast, prostate, and colon cancer cells. Silencing of the miR-200 family induces early transformation of epithelial cells leading to EMT, reinforcing the role of these miRNAs in tumor suppression [47].

An interesting link between the p53 tumor suppressor gene, SNAIL, and the miR-34 was described by Siemans et al. in 2011. In colon cancer cells, activation of p53 notably induced the expression of miR-34 family genes, resulting in the inhibition of SNAIL activation. As would be predicted, a correlate decrease in EMT markers was seen in the absence of SNAIL activation in this cell line. Treatment of cells with miR-34 halted TGFβ dependent EMT. It was noted, however, that the action of miR-34 family members was not unidirectional. The miR-34 family promoter sequences contain E-boxes which can be bound by SNAIL, resulting in decreased expression of miR-34. This double negative feedback loop between miR-34 and SNAIL offers an interesting regulatory mechanism for EMT creating unique challenges in drug development targeting either of these factors [48].

In contrast to the tumor suppressive functions of miR-200 and miR-34, the miR-9 family appears to be tumor activating through a direct interaction with the CDH1 promoter which subsequently initiates EMT [49]. miR-9 expression is increased in primary breast, gastric, and brain cancers [29]. Additionally, increased expression of miR-9 is seen in metastatic disease further supporting a tumorigenic role for this family of miRNAs.

It must be emphasized that work with non-coding RNAs is far from complete and their relevance to cancer (and cancer therapy) remains imprecise. While the miR-200 family appears to have compelling data for a tumor suppressive function, as discussed above, there is are also data indicating a pro-metastatic niche for miR-200 family members.

In samples from colon cancer patients, there is lowered expression of miR-200 within the primary tumor which fits the impression that miR-200 is tumor suppressive. However, in metastatic lesions, miR-200 expression is high [47],[50]. This allows for argument that miR-200 plays a pro-metastatic role in cancer by stabilizing the mesenchymal phenotype after deposition to distant sites (i.e. an important regulatory element in the mesenchymal to epithelial transition). Other evidence for a pro-metastatic role for miR-200 is seen in pancreatic, breast, and gastric cancer.

Another important miRNA family is the miR-10 group including miR10b. In the study of metastatic breast cancer cells, miR-10b was noted to be highly expressed and positively regulate cell invasion. It was also able to initiate tumor formation and played a role in distant metastases. Taken together, these properties are highly suggestive that miR-10b is implicated in EMT [51].

3. Cancer stem cells

The concept of the cancer stem cell is based on the hypothesis that a single cell type or subset of cells can act as tumor-initiators in various cancers ranging from hematopoietic to solid organ. By definition, these cancer stem cells (CSC) have features which give them “stem-like” properties allowing for self-renewal and differentiation leading to the histologic heterogeneity displayed by tumors [52].

Seminal work done in the mid-1990s provided compelling evidence supporting the role of CSCs in tumor initiation and progression in acute myeloid leukemia (AML). The AML-initiating cells, defined by their cell surface marker expression (CD34^+^,CD38^-^), were able to proliferate, disseminate, and maintain an AML phenotype similar to the patients they were derived from after transplantation into severe combined immune-deficient (SCID) mice [53].

Furthermore, the AML-initiating cells appeared to be derived from normal hematopoietic stem cells directly linking stem cells to tumorigenic cells. In other words, normal primitive cells underwent leukemic transformation resulting in the first experimentally described CSC population [54]. This pattern of tumor-initiation has subsequently been described in various solid tumors including brain, breast, pancreatic, prostate, and others [55]-[59].

Ultimately, CSCs exert their pathophysiologic impact in several ways. Their ability to self-renew and differentiate is at least partly responsible for the heterogeneity seen within tumors. One potential consequence of this is chemotherapy and radiotherapy resistance within populations of tumors. Additionally, CSCs can regenerate a tumor when transplanted into an appropriate host in experimental settings. This offers a proposed mechanism for metastatic disease and cancer recurrence. These properties have made CSCs, irrespective of their tumor of origin, extremely important for cancer therapy and an important new cancer target.

The machinery of EMT appears to connect directly to the formation and maintenance of CSCs. Mani et al. induced EMT in human, non-tumorigenic mammary epithelial cells using either TWIST or SNAIL transcription factors. As seen in prior studies, exposed cells acquired a mesenchymal phenotype [30]. Studying these cells using flow cytometry revealed a pattern of cell surface expression that was seen in breast cancer stem cells (CD44^High^/CD24^Low^). EMT made non-tumorigenic epithelial cells, at least in breast tissue, look like cancer stem cells.

While it was interesting that EMT made epithelial cells look like stem cells, more important was the discovery that these cells acted like stem cells. CD44^High^/CD24^Low^ cells were able to self-renew and give rise to multiple cell lineages. Furthermore, it was noted that CD44^High^/CD24^Low^ cells expressed a mesenchymal phenotype and up-regulated the expression of EMT transcription factors [60]. Together, these findings connected two important cancer-related processes – CSCs and EMT.

Other important EMT signaling pathways are also implicated in the transformation and maintenance of CSCs. For example, Wnt signaling is a well-studied pathway necessary for a variety of EMTs, both developmental and pathologic [61]. Wnt signaling is classically described as being canonical or non-canonical based on the involvement of β-catenin [62]. Hyperactivity of canonical and non-canonical Wnt pathways can trigger EMT programs and, more recently, play key roles in CSC biology [61].

In fact, Wnt activity may be used as a marker for defining CSCs in some malignancies. Using colon CSCs derived from patient samples, Wnt signaling was assessed using a GFP reporter system. Cells with high GFP expression notably had increased clonogenicity compared to those cells with low GFP expression. Stated another way, colon CSCs with high Wnt expression were highly potent at inducing tumors following injection into mice. The extent of Wnt activation was positively correlated with expression of well-studied cell surface makers for CSCs [63].

Notch signaling is another important pathway required for the conversion and maintenance of CSCs using EMT. Multiples studies show the importance of Notch signaling in multiple breast cancer subtypes and its role in invasive disease. Notch signaling, specifically Notch4 receptor activation, is increased in breast CSCs. In addition, Notch signal pathway inhibition decreases the number of breast CSCs both in vitro and in vivo [64].

The importance of Notch signaling is also seen in pancreatic CSCs. The γ-secretase inhibitor IX can be used to inactivate Notch signaling. After treating pancreatic CSCs with this secretase inhibitor, cell proliferation, migration, and invasion are all decreased. The mechanism for this appears to be through inhibiting the mesenchymal transition of pancreatic cells lines demonstrating the importance of Notch signaling to EMT [65]. Treatments directed at Wnt and Notch signal pathways may allow for regulation of EMT in tumors and modulation of CSCs.

A property of CSCs with important clinical relevance is the observation that they demonstrate resistance to both chemotherapy and radiotherapy [59]. Imatinib mesylate is a tyrosine kinase inhibitor used for the treatment of chronic myeloid leukemia (CML) with excellent clinical result. Early studies, following its approval for use by the Food and Drug Administration in 2001, showed a complete response to therapy in 76% of patients at a median follow-up of 19 months [66].

However, these results have since been somewhat tempered by the finding that patients with an early response to imatinib show evidence of disease recurrence [67]. The mechanism for this appears to be supported by a significant role for CSCs in resisting initial treatment and leading the progression to cancer recurrence as CSCs isolated from patients with CML show insensitivity to imatinib [68]. Some authors describe this as the “weed” or “dandelion” hypothesis as it may appear the problem is solved, but until the root is removed the weed can and will come back [69].

The “dandelion” hypothesis was tested by Li et al. in a breast cancer model. Cells were taken from patients with HER2+ breast cancer before and after treatment with lapatinib. Following chemotherapy, the percentage of CD44^High^/CD24^Low^ cells increased. These cells, with a molecular signature consistent with breast CSCs, were able to self-renew and had a propensity for tumor formation [70]. CD44^High^/CD24^Low^ breast CSCs appear to also show resistance to conventional radiation therapies [71]. The implication of these data are two-fold. First, chemotherapy and radiotherapy appear to select for the survival CSCs. Second, chemotherapy and radiotherapy do not impair the ability of CSCs to grow and replicate.

The focus of this review to this point has been on EMT, however an important process that must be discussed is its “opposite” pathway, mesenchymal to epithelial transition (MET). Just as EMT plays a critical role in development, MET also is a process first recognized in development. One specific example is the formation of epithelial nephrons which require the transcription factor Pax-2 to initiate MET. This illustrates that while MET functions to transition cells to an epithelial phenotype, the regulatory factors required are not necessarily the same for both processes [72]. MET is an expansive topic and the migrating cancer stem cell (MCSC) model will be discussed to briefly emphasize the role MET plays in metastatic disease and distant seeding of tumor cells.

The (MCSC) model attempts to integrate the concepts of CSC, EMT, MET, and metastatic disease. CSCs are generated via an EMT allowing them the ability to migrate and disseminate. Upon reaching a distant site, the CSC is exposed to a new environment which can either promote dormancy or undergo a MET to recapture epithelial characteristics. The next step would be the formation of a metastatic lesion with conserved features from the primary tumor [73].

The relevance to this model on a clinical level cannot be understated as therapies targeting EMT could inadvertently promote MET and progression of disease. Overcoming these challenges will likely define the next steps in cancer drug development directed at EMTs.

### 2.2 SECTION 2: Targeting EMT and developing novel therapies

Several mechanisms have been proposed to target EMT to develop novel cancer therapeutics. These targets include transcriptional regulators (ie SNAIL, TGFβ), non-coding RNAs, and CSCs. Additionally, a new focus of research centers on the interactions between the tumor microenvironment and its role in initiation, propagation, and termination of EMT. Novel therapeutic targets have emerged within the tumor microenvironment which have promising implications for cancer therapy.

As the key players within the epithelial-mesenchymal transition (EMT) continue to be elucidated, novel cancer targets are being identified within this framework to direct future clinical applications. Broadly, these therapeutic targets can be classified into 2 large categories: (1) preventing EMT (2) altering the tumor microenvironment.

#### 2.2.1 Preventing EMT

##### Targeting mediators of transcriptional control

The first target for preventing EMT is developing interventions against very specific intermediates of the transition. Key players, as discussed, include TGFβ and transcription factors, and most current clinically-applicable therapies are based on these targets. TGFβ activity is modulated with several strategies including small molecule inhibitors and exogenous compounds.

1. Small molecule inhibitors

TGFβ signaling has classically been targeted using antisense oligonucleotides, monoclonal antibodies, and chemical inhibitors for treatment of various inflammatory and neoplastic conditions [74]. Small molecule inhibitors offer a novel approach to modulating the effects of TGFβ at multiple levels [75].

CX-4945 is a small molecule inhibitor of CK2 (protein kinase CK2, a serine/threonine kinase) and is the first orally bioavailable inhibitor in its class. Previously, a link between CK2 and TGFβ signaling was proposed, specifically by inducing EMT [76]. Treatment of a human lung adenocarcinoma cell line (A549) with CX-4945 demonstrated a potent inhibition on the signaling of TGFβ and down-regulation of EMT markers. CX-4945 inhibited the TGFβ1 cadherin switch, preventing progression towards a mesenchymal phenotype by A549 cells. Additionally, CX-4945 halted TGFβ mediated signaling in both canonical Smad pathways and non-Smad pathways (Akt and ERK1/2), Wnt pathways, and focal adhesion pathways (FAK, Src, MMP, NF-kB) [77].

CX-4945 (Silmitasertib) is currently in clinical trial for the treatment of advanced solid tumors and multiple myeloma. It has reached phase 2 study for treatment of non-resectable cholangiocarcinoma in combination with gemcitabine and cisplatin compared to standard care of gemcitabine plus cisplatin. The study is presently active and recruiting patients. CX-4945 is also in phase 1 study for the treatment of relapsed or refractory multiple myeloma.

Another group of small molecules targeting TGFβ signaling are ALK5 kinase inhibitors. Several have been described including EW-7195, EW-7203, EW-7197, IN-1130, SB-431542, SD-208, SD-093, Ki26894, LY580276, LY-573636, LY-2109761, and LY2152799 (see Table 1) [78],[79]. Each are novel small molecule inhibitors of ALK5 kinase (or TGFβ type 1 receptor kinase), required for TGFβ 1 signaling.

**Table 1:** Small molecule inhibitors, mechanism, clinical targets, delivery, and status in clinical application

**Inhibitors**	**Mechanism**	**Target**	**Delivery**	**Clinical status**
CX-4945	CK2 inhibitor	Lung, myeloma	Oral, IV	Phase 1/2
EW-7195	TGFβ Inhibitor	Breast, Lung metastases	Orally available	Phase 1 Clinical Trial
EW-7203	TGFβ 1 and 2 receptor kinase inhibitor	Breast, Lung metastases	IV	Pre-Clinical
EW-7197	TGFβ 1 and 2 receptor kinase inhibitor	Breast, Lung metastases	IV	Pre-Clinical
IN-1130	TGFβ 1 and 2 receptor kinase inhibitor	Breast, Lung metastases	IV	Pre-Clinical
SB-431542	Inhibition of ATP binding to ALK5 kinase	Glioma	IV	Pre-Clinical
SD-208	TGFβ 1 receptor kinase inhibitor	Metastatic breast cancer	IV	Pre-Clinical
SD-093	TGFβ 1 receptor kinase inhibitor	Metastatic breast cancer	IV	Pre-Clinical
LY-2152799	TGFβ 1 receptor kinase antagonist	Breast, Myelodysplastic syndromes, Hepatocellular Carcinoma, Pancreatic Cancer, Malignant Glioma	Orally available	Clinical trial: Phase 2/3; Phase 2; Phase 1b/2; Phase 1b/2a
LGK974	Porcupine inhibitor (Wnt signaling)	Breast	Orally available	Phase 1 Clinical Trial
CWP232291	Wnt signaling inhibitor	Hematologic malignancies (AML)	Oral, IV	Phase 1 Clinical Trial
MK-0752	ϒ-secretase inhibitor	Cranial tumors, solid tumors	Oral, IV	Phase 1/2 Clinical Trial

TGFβ ligand binds to type 1 and type II receptor kinases on the cell surface bringing them together allowing for phosphorylation of receptor 1 kinase. Following phosphorylation, signaling proceeds in the cell through Smad proteins (a total of at least 8 unique Smad proteins) ultimately translocating to the nucleus and regulating the expression of target genes [80]. Blocking the receptor kinase at the cell surface is an attractive target for drug development and has shown promise in blocking EMT for therapeutic benefit [81].

The potential clinical applicability of EW-7195 was seen in preventing TGF1-β-dependent breast tumor metastasis to the lung. EW-7195 inhibited both TGFβ induced EMT and cell motility in vitro while inhibiting lung metastasis in vivo [82]. EW-7203, IN-1130, and EW-7197 were developed by the same group and demonstrate a similar function in halting the TGF1β-dependent EMT and also inhibiting metastatic spread of primary tumor (breast to lung) [82].

SB-431542 is another small molecule inhibitor exerting its affect through a highly specific interaction with ALK5 kinase. SB-431542 competitively inhibits binding to the ATP site of ALK5 kinase preventing downstream activation of TGFβ targets. It does not act on other kinase-dependent pathways including ERK, JNK, or p38 [83]. In a human glioma cell model, SB-431542 was able to reduce cell proliferation and motility by repressing the expression of EMT proteins [84]. Despite this promising data, SB-431542, developed by GlaxoSmithKline (GSK), has not made the transition to the clinical setting. Building on the work with SB-431542, an orally available small molecular inhibitor with similar mechanism of action, GW-788388 is currently being studied. GW-788388 has shown promise for application in halting the progression of renal fibrosis by inhibiting EMT [85]. Interestingly, GW-788388 also shows potential as a treatment for reversing cardiac fibrosis in patients with Chagas disease [86].

SD-208 and SD-093 are two additional small-molecule inhibitors which target TGFβ-dependent EMT. SD-208, an orally bioavailable compound, was noted to inhibit the development of pulmonary fibrosis in a rat model. Subsequently, SD-208 was used *in vivo* in mouse metastatic breast cancer models. SD-208 conferred a strong anti-tumor effect in treated mice with no obvious signs of toxicity. SD-208 remains in the pre-clinical phase at this time, however these data support a move to clinical trial in the near future [87].

More selective small molecule inhibitors targeting ALK5 kinase with specific activity towards either Smad or non-Smad pathways have also been studied for their ability to reverse or halt EMT in cancer progression. The ALK5 inhibitor A-83-01 appears to inhibit Smad signaling with little effect on non-canonical pathways. Similar to the other ALK5 small molecule inhibitors, A-83-01 demonstrates the ability to decrease expression of EMT markers in a TGFβ-dependent fashion. A-83-01 notably showed significantly more potency than other similar small molecule inhibitors. [88].

Another TGFβ small molecule inhibitor, LY2157299, has gained attention as a possible adjunctive treatment for chemotherapy resistant cancers. In the case of triple negative breast cancer, many are initially responsive to traditional chemotherapeutic agents; however, the likelihood of recurrence is high, typically with a more aggressive, less responsive tumor. One hypothesis for this pattern of disease is the resistance of a small population of cells within the initial tumor to traditional chemotherapy agents. This specialized group of cells, often described as being cancer stem cells, relies on mechanisms involving EMT to continue to survive [52].

Based on this background, LY2157299 (TGFβ kinase antagonist) was used to treat a population of triple negative breast cancer cells previously treated with the chemotherapeutic agent paclitaxel. LY2157299 treatment reduced the size of paclitaxel-resistant cells, slowed the growth rate of tumors, and reduced the tumor forming potential of residual cancer cells [89].

LY2157299, currently in the Lily Oncology pipeline, is being studied in clinical trial for application in myelodysplastic syndromes (phase 2/3), hepatocellular carcinoma (phase 2), unresectable pancreatic cancer (phase 1b/2), and malignant glioma (phase 1b/2a). The progression of LY2157299 into a potential clinical therapeutic highlights the potential of using small molecule inhibitors that target EMT to treat advances malignancies.

2. Exogenous compounds

Several exogenous compounds, including dietary and herbal chemopreventative agents may act in a TGFβ-dependent fashion to impair EMT. Resveratrol is a natural polyphenol found most commonly in grapes and red wine. It has been discussed in the oncology literature based on reports of its ability to suppress cancer metastasis and invasion [90],[91]. Many mechanisms have been suggested for its mode of action, however compelling data suggest resveratrol may inhibit EMT. In a lung cancer model (A549 cells), treatment with TGFβ alone compared with TGFβ and resveratrol showed no initiation of EMT in the latter group. Cells treated with resveratrol increased expression of epithelial markers while repressing fibronectin and vimentin. The mechanism appeared to rely on modulating EMT transcription factors SNAIL1 and SLUG [92].

Another dietary agent EGCG, the major polyphenolic compound in green tea, demonstrates anti-proliferative effects in several cancers [93]. EGCG inhibits the induction of p300/CBP, a histone acetyltransferase with known activity on Smad2 and Smad3. In lung cancer cells treated with EGCG, TGFβ induction of EMT was potently halted in a Smad2/Smad3-dependent fashion. EGCG, acting through p300/CBP was able to prevent EMT and blocked invasion of lung cancer cells [94].

The translation of bench findings to clinical trial for both resveratrol and EGCG has been challenging. At this stage, resveratrol has been studied in phase 1 clinical trial and EGCG remains in pre-clinical study. Other dietary and herbal chemopreventative agents also have been noted to inhibit EMT through non-TGFβ-dependent mechanisms. β-elemene, an active component of the herbal medicine Curcuma wenyujin, acts to stop EMT through Smad3 [95].

Curcumin, the active component of the spice turmeric, is an inhibitor of EMT by altering the SNAIL1 transcription factor [12]. 2-Hydroxycinnamaldehyde also inhibits EMT via a SNAIL-dependent mechanism in breast cancer cells and also prevents lung metastasis in a mouse orthotopic breast cancer model [96]. The next steps for each is devising the best mode of drug delivery, further drug development, and devising treatments to synergize with existing cancer therapeutics.

##### Non-coding RNAs

Studying miRNAs and their potential role in cancer is an extremely fast-growing area of research. In parallel, several research groups are working on ways to modulate the effect of miRNAs in tumor biology with the intent of developing new and novel therapeutic targets. Antagomirs and miRNA sponges each show promise for future clinical application and will be discussed.

1. Antagomirs

As described earlier, miRNAs can play a role in cancer progression and metastases leading to them often being referred to as “onco-miRs” [97]. Antagomirs were first described and designed by Krϋtzfeldt et al. in 2005. They were developed as pharmacologic agents to silence miRNAs in vivo and defined as being single-stranded RNAs complementary to miRNAs. Krϋtzfeldt’s group demonstrated antagomir-122, selective against miR-122, completely silenced its target in multiple tissues types [98]. Similar findings were seen with other antagomirs showing a broad bioavailability and potent effect across tissue types.

The same group responsible for determining the important role played by miR-10b in breast cancer invasion and metastatic progression also utilized an antagomir (antagomir-10b) to silence miR-10b in vitro and in vivo. Interestingly, antagomir-10b did not reduce the bulk or size of the primary mammary tumor. However, it significantly suppressed the formation of metastatic lesions in a mouse. Overall it was well tolerated by treated animals laying the groundwork for further study of antagomirs as anti-cancer drugs [51],[99].

2. miRNA sponges

miRNA sponges are another tool increasingly utilized to modify the activity of miRNAs [100]. These sponges are constructed by utilizing 3’UTR mRNA sites complementary to targeted miRNA. By doing so, they act as a binding site for specific miRNAs and functionally inhibit miRNA binding to native targets [101]. Early work demonstrates that miRNA sponges can down-regulate tumorigenic miRNAs in vivo [102]. The important question that remains to be answered is how well this strategy would work as a cancer therapy and if it would be tolerated by patients.

As illustrated, this remains a relatively young area of work with few advancements to the clinical setting to date. However, strategies including the development of antagomirs and miRNA sponges have the potential to make an impact on a patient level.

##### Cancer stem cells

As discussed, EMT signaling pathways have important roles in the transformation of epithelial cells into CSCs. Several groups have started the process towards drug development to inhibit this process using large-scale screening techniques [103],[104]. The results of these screens allows for the identification of potential pharmacologic agents for eliminating CSCs. Understanding the properties of these agents and assessing their biologic activity is the first step towards drug development as stand-alone agents or as adjunctive therapies [103].

Another strategy for eliminating CSCs is specifically targeting EMT pathways important for their generation and maintenance. Physiologic factors already exist to prevent mesenchymal transition, likely secreted by epithelial cells in order to remain in a fixed state. These autocrine factors include Dickkopf-1 (DKK1) and secreted frizzle-related protein 1 (SFRP1), both known inhibitors of Wnt pathways. These proteins could be pharmacologically mimicked to produce a similar effect in diseased states [105].

1. Small molecule inhibitors

Wnt pathway inhibitors currently in development for the purpose of inhibiting CSC formation include LGK974 and CWP232291 (see Table 1). LGK974 is a small molecule inhibitor of Porcupine, a Wnt-specific acyltransferase, and strongly inhibits Wnt signaling both in vitro and in vivo. In rodent breast cancer models, LGK974 demonstrated dose dependent tumor regression with acceptable tolerance and oral bioavailability [106]. LGK974 is presently in phase 1 clinical trial. CWP232291 is another small molecule Wnt pathway inhibitor which notably produced marked cytotoxicity against CSCs in hematologic malignancies while showing minimal effect on normal bone marrow cells. These findings led to its application for treating AML. CWP232291 is currently in recruitment for a phase 1 clinical trial for treatment of relapsed or recurrent AML [107].

Notch signaling has also been targeted with small molecule inhibitors, both as independent and adjunctive therapies. MK-0752 is a potent γ-secretase inhibitor with strong efficacy for regulating Notch signaling, particularly in cells with stem-like properties. MK-0752 is also orally available making it an attractive potential chemotherapeutic. Phase 1 studies show acceptable tolerability, however low efficacy as a single treatment for extra-cranial tumors [108]. MK-0752 is presently in clinical trial assessing its potential for use in multidrug regimens for advanced solid tumors.

2. Metformin

Small molecule inhibitors offer the potential for future therapeutic options against difficult to treat malignancies, particularly those which demonstrate treatment resistance. The transition from the bench to wide-spread use, however, is often long and difficult. Utilizing pharmacologic agents already used in clinical practice to target CSCs would have the potential to more quickly impact patients. Recent work with the oral hypoglycemic metformin offers a possible model to follow for applying this strategy. Metformin, a mainstay in contemporary management of type 2 diabetes mellitus, is extremely well tolerated and extensively used. The link between cancer and metformin was first reported in 2005 after studying a large group of patients using a national database [109].

Based on this observation, several groups tested the biologic impact of metformin on tumor progression in mice models. Interestingly, metformin can inhibit the growth of solid tumors, including breast tumors, in both diabetic and non-diabetic mice [110],[111]. Several mechanisms have been proposed to explain this result, however it appears that metformin can selectively inhibit the growth of breast CSCs by modifying the EMT machinery [112]. When combined with conventional chemotherapeutics, the effect is potentiated [113]. This is an important example of leveraging existing therapies to modify EMTs. Additional EMT pathway targets and novel pharmacologic agents targeting CSCs are well-summarized in another recent review [114].

#### 2.2.2 Altering the tumor microenvironment

To this point, EMT has largely been discussed as a process at the tumor level, with the transition to a mesenchymal phenotype occurring in tightly regulated fashion. Inducers of EMT come from many sources including the tissue environment in which the cell originates. With travel to distant sites, as is the case in metastatic disease, a new microenvironment in encountered. Clearly, the interplay between the tumor microenvironment (TMEN) and the tumor – including the microenvironment as a stimulator of EMTs – is important in cancer progression [115]. Targeting and altering the TMEN offers another approach to direct therapies to prevent EMT.

##### Components of the TMEN

The TMEN is a specialized extracellular matrix (ECM) wherein a cancer cell proliferates [116]. The components, therefore, are similar to those seen in any inflammatory ECM and include soluble factors, cytokines, chemokines, neovascular elements, immune cells, and mesenchymal stem cells (and their derivatives) [117],[118] Within the microenvironment, several components are clearly cancer-promoting elements [119].

The cytokines IL-1, IL-8, IL-10, and TGFβ all demonstrate effects on either tumor growth or metastasis through their interactions with cancer cells. The major cell types responsible for these mediators in the TMEN include the primary tumor cell and stromal cells including macrophages, adipocytes, and cancer associated fibroblasts (CAFs) [118]. A key cell type in the TMEN are mesenchymal stem cells (MSCs) which exhibit the ability to differentiate into nearly all stromal cell lineages. The plasticity of MSCs and their importance in maintaining the TMEN make them a critical target in cancer therapy [120].

##### Microenvironmental regulators of EMT

Mesenchymal stem cells (MSCs) have the ability to induce EMTs in several models and play a role in both pre-metastatic and metastatic sites. Breast cancer cell lines (MDA-MB-231, T47D, SK-Br3) cultured with MSCs increase expression of EMT markers (N-cadherin, Vimentin, Twist, SNAIL) with concomitant decrease in E-cadherin expression. This increase in EMT markers corresponded with an increase in protein expression as well, with increased Vimentin and SNAIL compared to untreated cells. Gene expression assays also revealed upregulation of TGFβ-receptor implicating TGFβ mechanistically as a regulator of this EMT. This occurred with only co-culturing these breast cancer cell lines with MSCs and no further co-stimulatory factors [121].

A similar role for MSCs initiating EMT is seen in hepatocellular carcinoma (HCC). HCC cell lines (SMMC-7721 and Hep-3B) expressed EMT markers after exposure to IFNy and TNF-alpha stimulated MSCs. The mechanism of action appeared to be via TGFβ expressed by MSCs, similar to results seen in breast cancer cell lines. These findings were correlated to clinical samples revealing over-expression of SSEA-4 (a marker for MSCs in clinical HCC tissues) was found to confer a shorter cancer-free interval and worse overall survival [122].

While MSCs have the ability to initiate EMTs, they also differentiate into other cell types within the TMEN to potentiate their action as tumor promoters. One particular cell type, the cancer-associated fibroblast (CAF) has garnered significant attention as a target for cancer therapy [123]. CAFs are defined by their similarities with myofibroblasts based on their origin (bone marrow or by EMT of resident fibroblasts) and pattern of surface expression (most notably alpha-SMA, MMP1, MMP3) [124]. Unlike myofibroblasts, CAFs are not removed by apoptosis and cannot be “deactivated”. Given that CAFs are the most abundant cell type in the TMEN in several cancers (breast, prostate, pancreatic), that they can stimulate EMT, and they are important mediators of chemotherapy resistance, eliminating CAFs offers an exciting drug target.

##### Therapeutic targets of TMEN-stimulated EMTs

The most significant challenge of designing therapies to eliminate CAFs are their similarities with myofibroblasts – which are essential for wound healing. Predictably, the pathways used in cancer progression are also essential for wound healing [125]. Osteopontin, a secreted phosphoprotein implicated in tumor metastasis both mechanistically and as a marker, is able to induce MSCs to adopt a CAF phenotype through autocrine signaling of TGFβ-1 [126]. This pathway, first observed in breast cancer, was also noted to be conserved in bone fracture healing (unpublished data). Unlike in cancer, where this signaling pathway is pathologic, fracture healing is dependent on the differentiation of MSCs into myofibroblasts to ensure appropriate bone remodeling. Cancer drugs must be specific to CAFs without negatively affecting myofibroblasts or other cell lines important for healing.

Despite these inherent challenges, strategies to inhibit CAFs have been tried at numerous potential points of intervention. Imatinib mesylate, a tyrosine kinase inhibitor currently used in the clinical setting for treatment of numerous malignancies, decreased the viability of CAFs in vitro by inhibiting expression of alpha-SMA [127]. Another approach targeting TGFβ uses the antisense oligonucleotide AP-12009 for the treatment of glioma [128]. At present, AP-12009 (Trabedersen) is being prepared to be tested in a phase II clinical trial for systemic IV administration in malignant melanoma and pancreatic cancer.

Another potential site for intervention within the TMEN include blocking the interaction between MSCs and cancer cells by preventing MSC recruitment to tumor sites. Zoledronic acid (ZA), a bisphosphonate, appears to show promise in this role. Specifically, in a breast cancer model, treatment with ZA prevented MSC migration by decreasing the expression of RANTES and IL-6, both markers for MSC migration. These data suggest a role for ZA as an adjunctive therapy to conventional chemotherapeutics [129].

## 3 Conclusions

EMT plays a considerable role both in normal development and diseased states. Understanding the interplay between EMT and development, both embryologically and in wound healing, has offered unique perspectives in determining the key regulatory steps for progression to disease. Shared molecular pathways amongst all EMTs allows for the development of novel therapies to potentially halt or reverse EMT prior to metastatic spread. Small molecule inhibitors and exogenous compounds appear to offer the most promise at this stage.

Additionally, the interplay between the TMEN and induction of EMT creates yet another opportunity to design targeted therapies in cancer. Modulating the key effectors within the tumor stroma is an area ripe for drug development. Undoubtedly, EMTs and their regulators have significant clinical implication across the oncologic spectrum.

## Abbreviations

EMT: Epithelial-mesenchymal transition

TGF: Transforming growth factor

EGFR: Epidermal growth factor receptor

ECM: Extracellular matrix

IL: Interleukin

MAPK: Mitogen-activated protein kinase

nAChRs: Nicotinic acetylcholine receptor

ROS: Reactive oxygen species

miR: micro-RNA

ALK5: TGFβ type 1 receptor

CK2: Protein kinase 2

INF: Interferon

## Competing interests

The authors declare that they have no competing interests.

## Authors’ contribution

AK and PK conceived the format for the review and participated in its design and drafting. Literature and data acquisition were conducted by AK. Final manuscript editing and review were conducting by PK. All authors read and approved the final manuscript.
